# Research Trends on Pulmonary Rehabilitation: A Bibliometric Analysis From 2011 to 2020

**DOI:** 10.3389/fmed.2022.887793

**Published:** 2022-06-06

**Authors:** Tao Li, Jia Chen

**Affiliations:** ^1^Department of Rehabilitation, Wenjiang Area Hospital of Traditional Chinese Medicine, Chengdu, China; ^2^Department of Traditional Chinese Medicine, Guangdong Pharmaceutical University, Guangzhou, China

**Keywords:** pulmonary rehabilitation, bibliometric analysis, research trends, CiteSpace, VOSviewer

## Abstract

**Background and Objective:**

A mounting body of evidence suggests that lung function may deteriorate over time with the development of chronic lung diseases (CRDs). Pulmonary rehabilitation has been proved to improve exercise capacity and quality of life in individuals with CRDs. However, PR remains grossly underutilized all around the world. This study aimed to analyze the research trends on PR over the past 10 years.

**Methods:**

The publications related to pulmonary rehabilitation in the Web of Science Core Collection (WoSCC) from 2011 to 2020 were searched. VOSviewer (1.6.15) and CiteSpace Software (5.5.R2) were used to analyze authors and co-cited authors, countries and institutions, journals and co-cited journals, co-cited references, and keywords.

**Results:**

A total of 4,521 publications were retrieved between 2011 and 2020, and the number of annual publications on pulmonary rehabilitation has shown an overall upward trend in the past decade. The USA was the most productive country, the University of Toronto from Canada was both the first in publications and citations. Spruit MA was both the most productive author and the one with the highest number of co-citations. The first productive journal was the International Journal of Chronic Obstructive Pulmonary Disease, while the first co-cited journal was the American Journal of Respiratory and Critical Care Medicine. The hot keywords were grouped into three clusters, while “Asthma” and “Respiratory society statement” were determined as the frontier topics.

**Conclusions:**

The present study successfully revealed the research status and development trends of pulmonary rehabilitation from 2011 to 2020 by using bibliometric analysis, which may help researchers explore and discover new research directions in the future.

## Introduction

Chronic respiratory diseases (CRDs), as the most common non-communicable diseases, are prevalent worldwide and affect both developed and developing countries (1), Research showed that CRDs are accounting for >10% of the global burden of disease (2), and in 2017, about 545 million people across the world had a CRD, among which global prevalence of chronic obstructive pulmonary disease (COPD) and asthma were 3.9 and 3.6%, respectively (3). In 2020, there were about 2.2 million new lung cancer cases and 1.8 million deaths worldwide (4). Although drug therapy and targeted therapy for these diseases are constantly applied, compared with 1990, the prevalence of CRDs worldwide in 2017 was still representing an increase of 39.8%, and the prevalence and mortality of CRDs are still at a high level (5). Therefore, preventing the occurrence and recurrence of CRD remains a huge challenge for the public health systems all over the world. Hopefully, after decades of clinical confirmation, pulmonary rehabilitation (PR) has been suggested as an effective and non-invasive intervention for patients with CRDs. As an interdisciplinary intervention, PR is designed to improve the physical status and the psychological condition of individuals with CRDs, including improving quality of life, exercise capacity, and dyspnea-relief (6, 7). Recommendations have also been published by the American Association of Cardiovascular and Pulmonary Rehabilitation (AACVPR), the American Thoracic Society (ATS), and the European Respiratory Society (ERS) (8, 9).

Despite those benefits mentioned above and recommendations by authorities, PR remains grossly underutilized all around the world (10), likely reasons include insufficient funding; insufficient allocation of health care expenditures for PR; lack of awareness of the benefits of PR; limited resources for PR programs; and lack of training opportunities for PR professionals (9). Thus, an accurate overview of the research status, trends, and frontiers are indispensable for understanding the influence of PR. In this present study, we made a bibliometric analysis of related publications by bibliometric tools to look into research trends and frontiers in the research area of PR over the past decade. Bibliometric analysis is nowadays gaining more popularity in various fields, which uses the characteristics of literature metrology to quantitively measure the inter-relationships, distribution characteristics, impacts of publications, and research trends in a certain field (11). With the help of bibliometric tools, such as CiteSpace, hotspots, and frontiers in a research field can be visualized and identified by analyzing co-authorship, co-citation, and co-occurrence (12).

In this present study, in order to seek the current worldwide research status and development trends on PR over the past 10 years, a bibliometric analysis was applied to analyze the related publications derived from the Web of Science Core Collection (WoSCC).

## Materials and Methods

### Data Acquisition and Search Query Strategy

**Figure 1:**
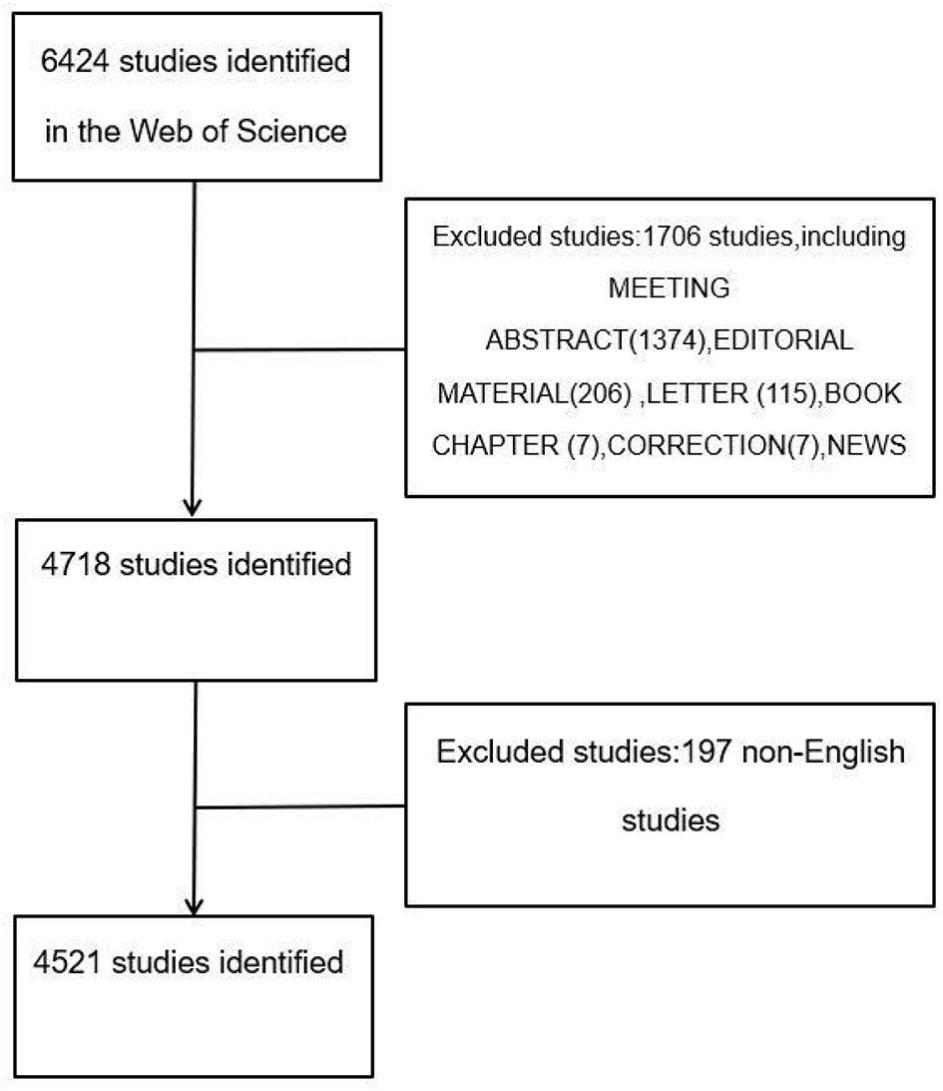
Flowchart of literature selection.

All research data in accordance with the present study were searched from WoSCC. The data was downloaded on October 9, 2021. The search strategy focused only on the published literature ranging from 2011 to 2020 with the following formula: Index = science citation index expanded (SCI-Expanded), TS = (Pulmonary rehabilitation) OR (Lung rehabilitation), timespan = 2011–2020. The whole process of data acquisition was conducted independently by two reviewers (TL and JC), and any discrepancy was resolved by consultation with each other. A total of 6,424 articles were retrieved, and document types incorporated into the present study were restricted to only articles and reviews. A total of 1,903 irrelevant articles (including meeting abstracts, editorial materials, letters, book chapters, corrections, news items, reprints, and non-English studies) were excluded (Figure 1). Ultimately, a total of 4,521 studies were included and exported in the form of all records and references, and then saved as plain text file documents and with the nam format “download_XX.txt.”

### Data Analysis

A bibliometric analysis by using VOSviewer (1.6.15) and CiteSpace (5.5.R2) was used to analyze all selected data. VOSviewer (13) was applied to build visual maps and summarize relevant information on authors, geographical distribution, journals, keywords, and references. CiteSpace (14) was used to analyze the burst co-cited references and keywords. The specific parameters of VOSviewer accepted the default settings itself, while CiteSpace software was set as the following steps: (1) Time Slicing: 2011 to 2020, selecting “1” as Years Per Slice; (2) Text processing: selecting “Title,” “Abstract,” “Author keywords,” and “Keywords Plus” as well; (3) Node type: a single node type; (4) Selection criteria: The most cited or occurred items from each slice were selected top 50 levels; (5) Pruning: selecting “pathfinder.” Furthermore, journal impact factors (IF) were identified according to the 2020 Journal Citation Reports (JCR 2020) published by Clarivate Analytics.

## Results

### Annual Publications

**Figure 2:**
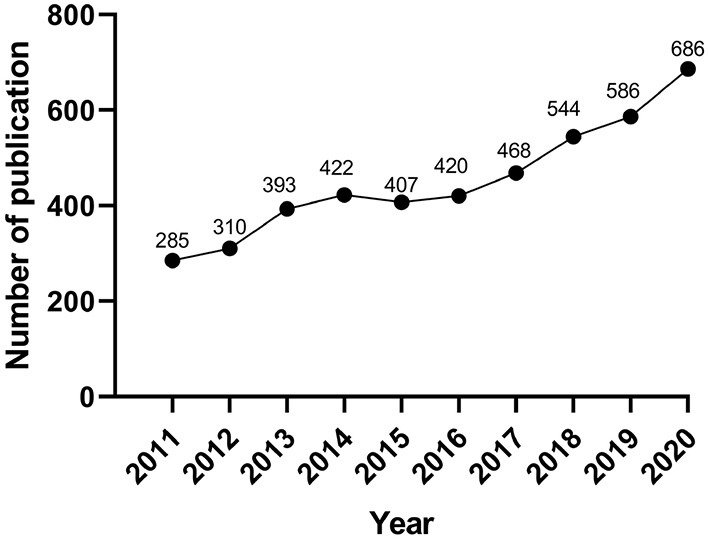
The annual number of publications on pulmonary rehabilitation between 2011 and 2020.

The number of annual publications can reflect the change of domain to a certain extent. In the present study, a total of 4,521 articles were finally identified according to the above retrieval method. The number of annual publications of research on PR is shown in Figure 2. We can visually observe that the amount of PR-related literature has increased constantly from 2011 to 2020 (except for a slight decrease in 2015), and the number of publications has reached its peak in 2020 (*n* = 686).

### Analysis of Authors and Co-cited Authors

**Figure 3:**
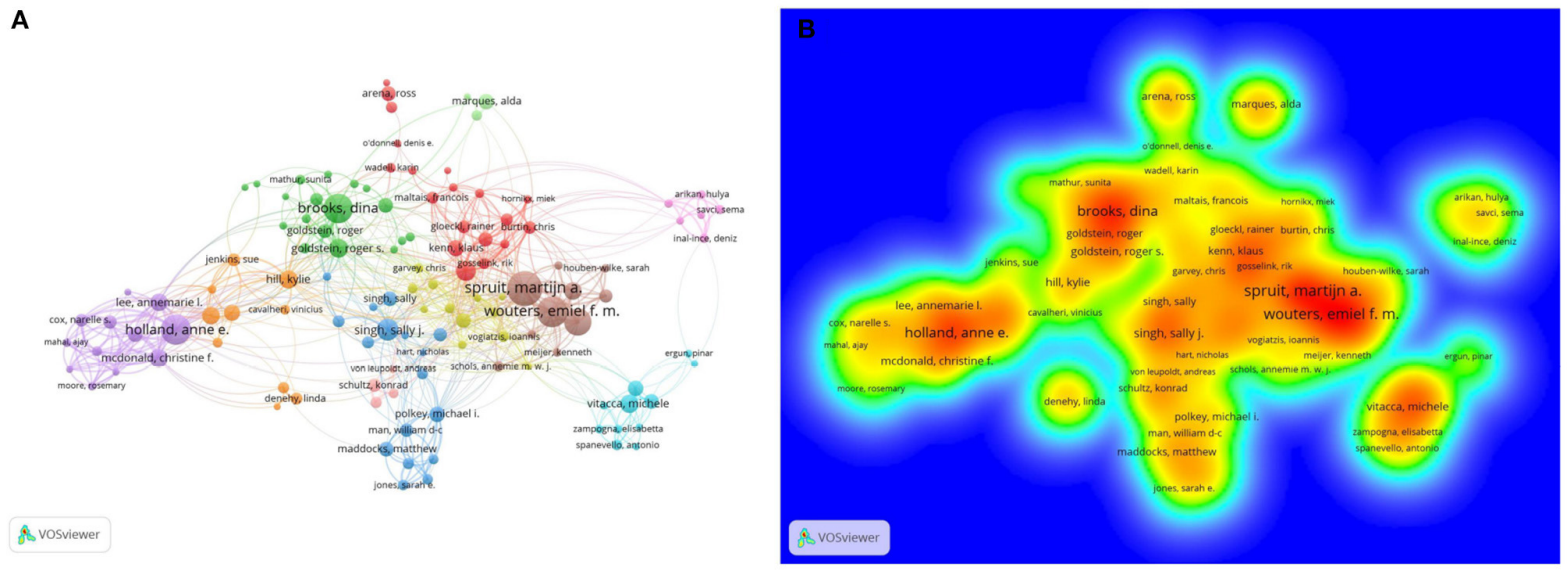
**(A)** VOSviewer visualization map co-occurrence of authors devoted to research on pulmonary rehabilitation. **(B)** The density map of co-cited authors. The closer the keyword node color is to red, the higher the frequency of its co-occurrence.

In total 19,487 authors were involved in research on PR, and 145 authors published more than 10 articles. Spruit MA ranked first among the prolific authors (*n* = 92), followed by Wouters EM (*n* = 77), Holland AE (*n* = 73), Brookes D (*n* = 71), and Franssen FE (*n* = 65). The remaining five authors had published 36–46 publications, respectively (Table 1). The authors (*n* = 145) who published at least 10 papers met the threshold (T ≥ 10, T = threshold) and were identified to construct the co-occurrence map of authors (Figure 3A), and these active authors formed 11 clusters. The same color represented the same cluster. Links between authors suggested that there were active collaborations in research on PR, especially among authors in the same cluster.

**Table 1:** Top 10 authors and co-cited authors.

**Author**	**Count**	**Co-cited author**	**Citation**
Spruit MA	92	Spruit MA	1,499
Wouters EM	77	Jones PW	890
Holland AE	73	Holland AE	805
Brookes D	71	Troosters T	714
Franssen FE	65	Ries AL	705
Singh SJ	46	Puhan MA	687
Troosters T	41	Crapo RO	666
Goldstein RS	38	Nici L	576
Mcdonald CF	37	O'donnell DE	549
Hill K	36	Pitta F	539

Co-cited authors are authors who have been co-cited together in a range of publications (15). Among a total of 63,565 co-cited authors, there were 149 authors co-cited over 100 times. Spruit MA (*n* = 1,499) ranked first, followed by Jones PW (*n* = 890), Holland AE (*n* = 805), Troosters T (*n* = 714), Ries AL (*n* = 705), and Puhan MA (*n* = 687). The last four top authors were co-cited with citations from 539 to 666 (Table 1). The authors (*n* = 29) with citations greater than 100 (T ≥ 100) were used to build the density map (Figure 3B), which could intuitively display the high-frequency co-cited authors clearly. As shown in Figure 3B, the nodes centered on Spruit MA had the hottest color among the co-cited authors.

### Distribution of Countries and Regions

**Figure 4:**
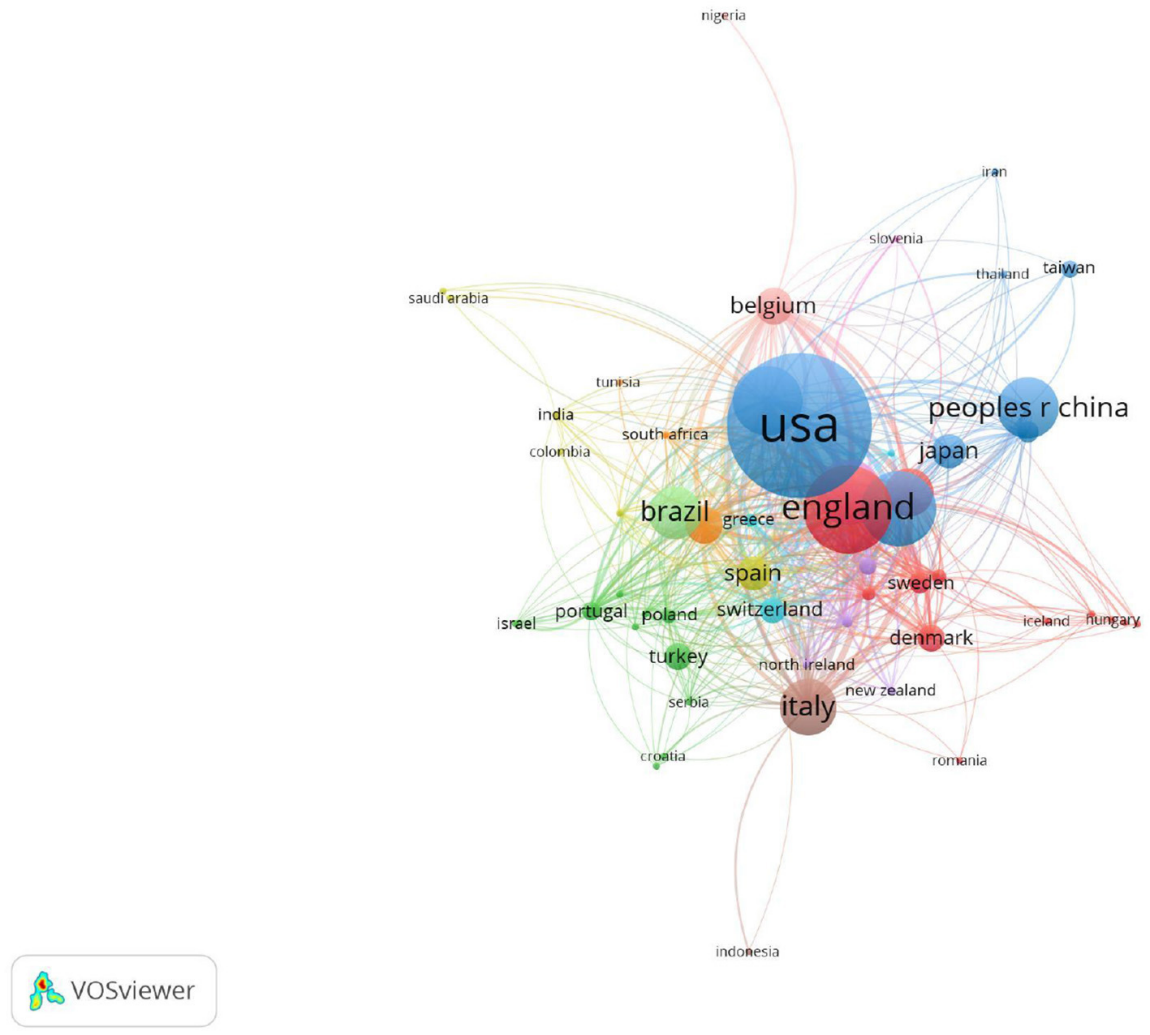
The co-authorship map of the distribution of country/region for the research on pulmonary rehabilitation.

According to the statistical analysis, 4,521 publications in total were co-authored by 5,401 institutions from 98 countries/regions, and there were 55 countries/regions that published more than five articles (Figure 4). Table 2 lists the top 10 most prolific countries and regions. Obviously, the first country with the highest number of publications was the USA (1,001, 22.14%), followed by England (539, 11.92%), Australia (442, 9.77%), Canada (406, 8.98%), and the Netherlands (371,8.21%). It is worth noting that the USA was not only ranked first in publications but also the first in citations in the field of PR.

**Table 2:** Publications and citations of countries/regions related to pulmonary rehabilitation.

**Rank**	**Country/** **region**	**Publications**	**Citations**	**Proportion (%)**
1	USA	1,001	24,169	22.14
2	England	539	18,002	11.92
3	Australia	442	10,996	9.77
4	Canada	406	11,037	8.98
5	Netherland	371	10,770	8.21
6	China	346	4,278	7.65
7	Italy	317	7,830	7.01
8	Brazil	281	4,023	6.21
9	Germany	259	8,158	6.09
10	Belgium	188	5,118	4.15

### Institutions

**Figure 5:**
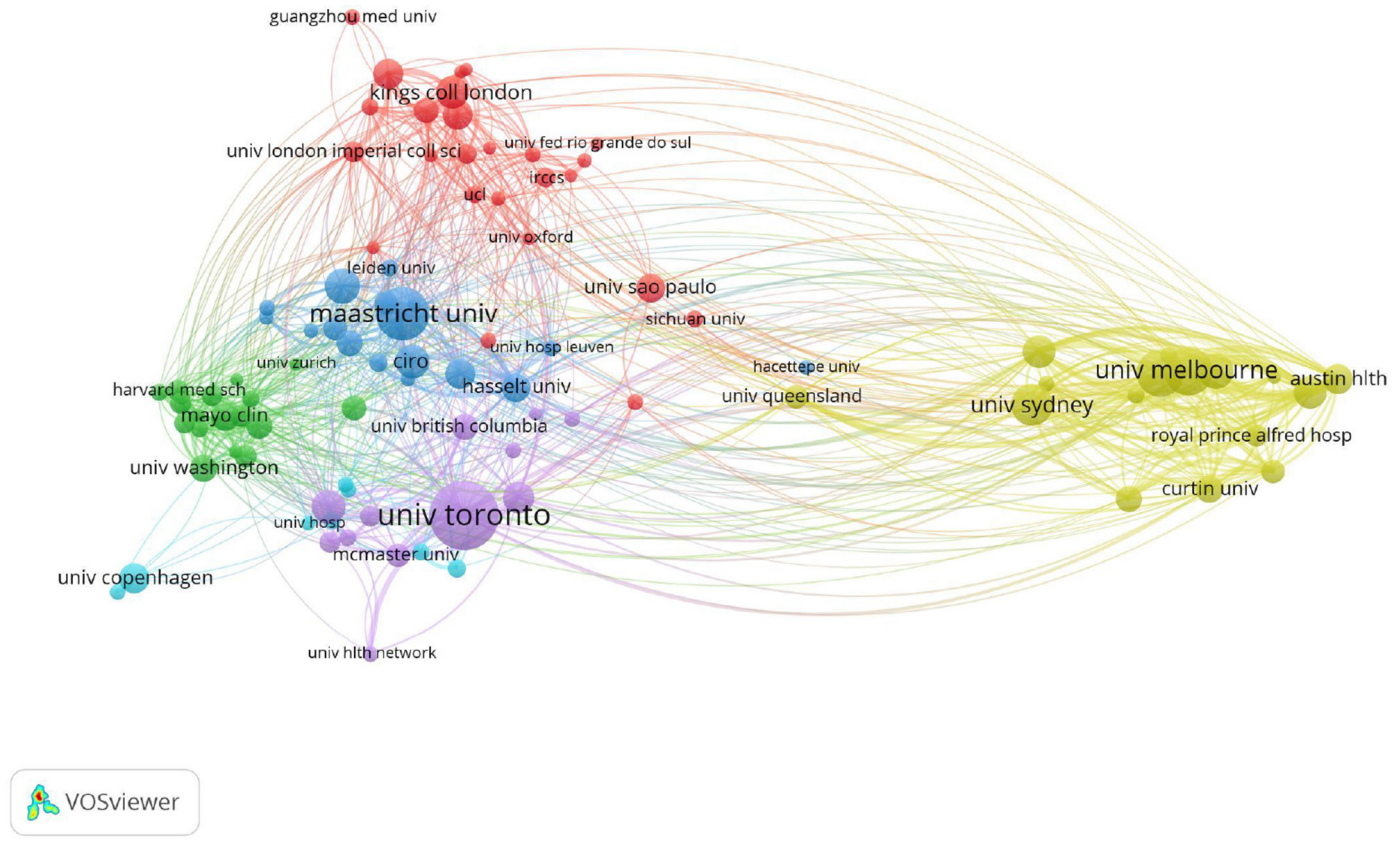
The co-authorship network map of institutions for the research on pulmonary rehabilitation.

In total 4,521 articles were published by 5,401 different institutions, and 96 institutions met the thresholds (T ≥ 20). The co-occurrence relations are shown in Figure 5, and we listed the top 10 prolific institutions in Table 3. The most prolific institution was University of Toronto (*n* = 150), followed by Maastricht University (*n* = 112), University of Melbourne (*n* = 96), La Trobe University (*n* = 93), and the University of Sydney (*n* = 81). The Institute for Breathing & Sleep, University of Groningen, Ciro, Mcgill University, and Monash University ranked 6th−10th, respectively.

**Table 3:** Top 10 publications of institutions related to pulmonary rehabilitation.

**Rank**	**Institution**	**Publications**	**Citations**	**Country**
1	University of Toronto	150	4,141	Canada
2	Maastricht University	112	2,409	Netherlands
3	University of Melbourne	96	2,885	Australia
4	La Trobe University	93	2,733	Australia
5	University of Sydney	81	1,465	Australia
6	Institute for Breathing & Sleep	66	1,555	Australia
7	University of Groningen	65	1,788	Netherlands
8	Ciro	64	1,265	Netherlands
9	Mcgill University	64	1,651	Canada
10	Monash University	61	1,014	Australia

### Analysis of Journals and Co-cited Journals

**Figure 6:**
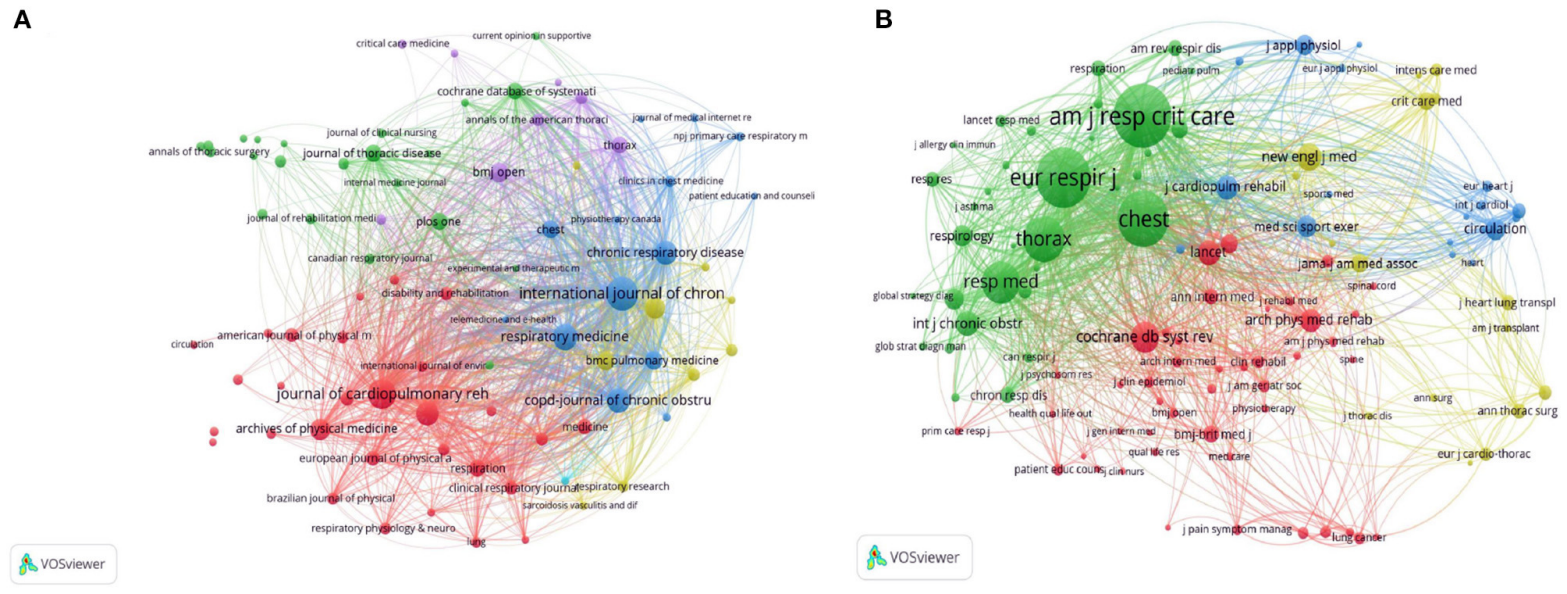
**(A)** The network map of academic journals [**(A)**, T ≥ 10]. **(B)** The network map of co-cited journals [**(B)**, T ≥ 200] for research on pulmonary rehabilitation.

All involved articles were published in a total of 917 journals, in which there were 83 journals that published more than 10 articles (Figure 6A). The IF and journal quartile were obtained from JCR 2020. As shown in Table 4, the three most prolific journals were the International Journal of Chronic Obstructive Pulmonary Disease (*n* = 209, IF = 3.55), the Journal of Cardiopulmonary Rehabilitation and Prevention (*n* = 162, IF = 2.081), and Respiratory Medicine (*n* = 119, IF = 3.145). In addition, the top five most prolific journals listed in Table 4 were mainly classified in Q2 or Q3, and their self-citation rate (SCR) was high, up to 10.67%. On the other hand, there were a total of 15,817 co-cited journals, among which 125 met the thresholds (T ≥ 200) (Figure 6B). The top three co-cited journals were American Journal of Respiratory and Critical Care Medicine (IF = 21.405), European Respiratory Journal (IF = 16.671), and Chest (IF = 9.41), and their SCR were <4%.

**Table 4:** Top 10 scholarly journals and co-cited journals related to research on pulmonary rehabilitation.

**Rank**	**Journal**	**Publications**	**IF (2020)**	**Self-citation rates (2020)**	**Rank**	**Co-cited journal**	**Co-citation**	**IF (2020)**	**Self-citation** **rates (2020)**
1	International Journal of Chronic Obstructive Pulmonary Disease	209	3.355 (Q2)	10.67%	1	American Journal of Respiratory and Critical Care Medicine	11,399	21.405 (Q1)	3.23%
2	Journal of Cardiopulmonary Rehabilitation and Prevention	162	2.081 (Q3)	9.68%	2	European Respiratory Journal	10,146	16.671 (Q1)	3.70%
3	Respiratory Medicine	119	3.415 (Q2)	2.22%	3	Chest	9,414	9.41 (Q1)	2.81%
4	COPD—Journal of Chronic Obstructive Pulmonary Disease	110	2.409 (Q4)	3.18%	4	Thorax	6,642	9.139 (Q1)	1.02%
5	Respiratory Care	105	2.258 (Q3)	8.13%	5	Respiratory Medicine	5,627	3.415 (Q2)	2.22%
6	Chronic Respiratory Disease	103	2.444 (Q4)	1.27%	6	Cochrane Database of Systematic Reviews	3,278	9.266 (Q1)	3.20%
7	Archives of Physical Medicine and Rehabilitation	82	3.966 (Q1)	2.60%	7	New England Journal of Medicine	2,793	91.245 (Q1)	0.04%
8	Respirology	81	6.424 (Q1)	5.01%	8	Journal of Cardiopulmonary Rehabilitation and Prevention	2,340	2.081 (Q3)	9.68%
9	BMJ Open	70	2.692 (Q2)	3.72%	9	Lancet	2,321	79.321 (Q1)	0.57%
10	BMC Pulmonary Medicine	65	3.317 (Q2)	2.20%	10	Archives of Physical Medicine and Rehabilitation	2,183	3.966 (Q1)	2.60%

### Co-cited References and Reference Burst

**Figure 7:**
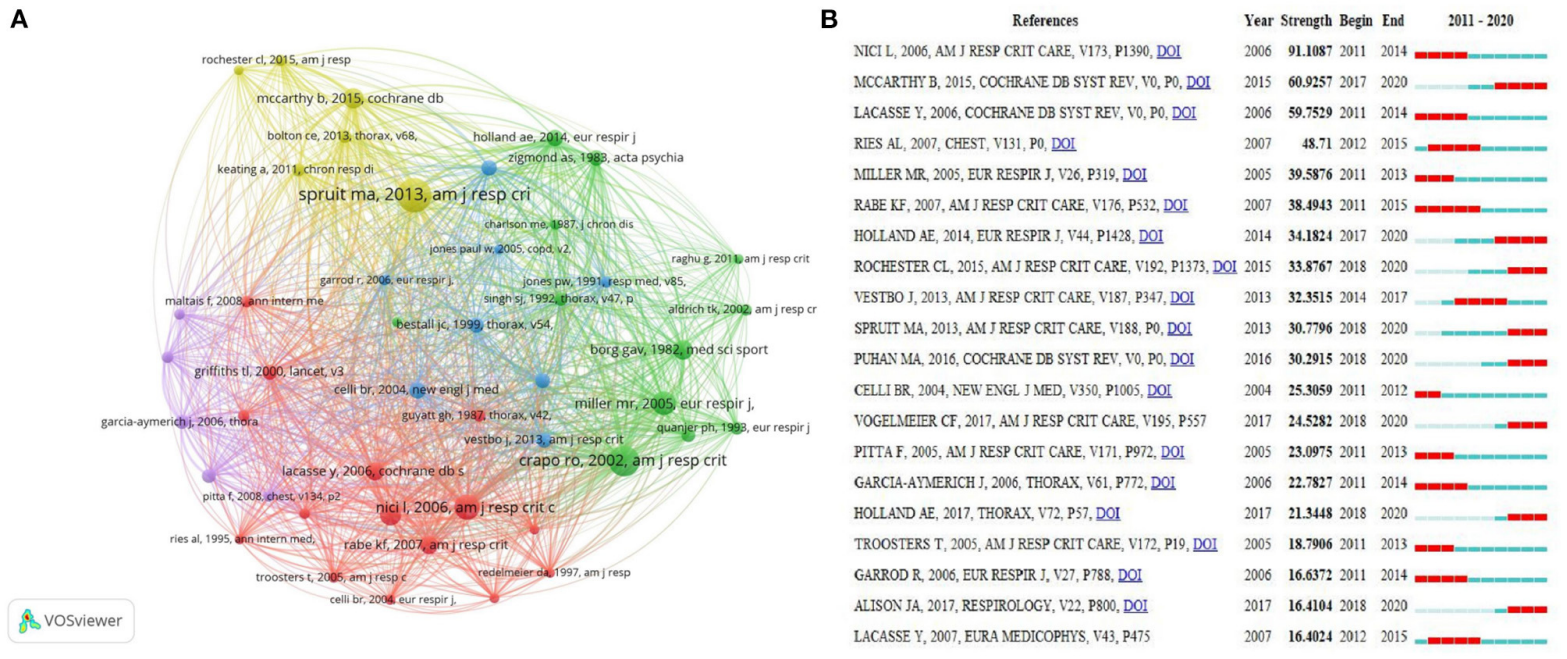
**(A)** The network map of co-cited references for research on pulmonary rehabilitation (T ≥ 100). **(B)** Top 20 References with the Strongest Citation Bursts on pulmonary rehabilitation.

Co-cited references are references that have been co-cited in a series of publications. According to VOSviewer analysis, 1,00,372 references in total were co-cited by all 4,521 publications, as shown in Figure 7A; 46 references with high co-citation met the thresholds (T ≥ 100). The top 10 co-cited references are listed in Table 5. The study entitled “An official American Thoracic Society/European Respiratory Society statement: key concepts and advances in pulmonary rehabilitation” (16) published in American Journal of Respiratory and Critical Care Medicine by Spruit et al. ranked first with 781 co-citations, followed by Crapo et al. (17) in the American Journal of Respiratory and Critical Care Medicine. (*n* = 627), Nici et al. (18) in the American Journal of Respiratory and Critical Care Medicine (*n* = 472), Miller et al. (19) in the European Respiratory Journal (*n* = 427), and Ries et al. (20) in Chest (*n* = 361). The next five references (21–25) had co-citations ranging from 240 to 335. In addition, these top 10 references were mainly divided into three types including guideline, review and symposium article, moreover, top five references were all guidelines.

**Table 5:** Top 10 co-cited reference with high citations related to research on pulmonary rehabilitation.

**Rank**	**Co-cited reference**	**Co-citations**	**Type**
1	Spruit MA. (2013). An official American thoracic society/European respiratory society statement: key concepts and advances in pulmonary rehabilitation. Am J Respir Crit Care Med. 188(8):e13–64. (16)	781	Guideline
2	Crapo R.O. (2002). ATS statement: guidelines for the 6-min walk test. Am J Respir Crit Care Med. 166(1):111–7. (17)	627	Guideline
3	Nici L. (2006). American thoracic society/European respiratory society statement on pulmonary rehabilitation. Am J Respir Crit Care Med. 173: 1390–1414. (18)	472	Guideline
4	Miller, MR. (2005). Standardization of spirometry. Eur Respir J. 26(2), 319–338. (19)	427	Guideline
5	Ries AL. (2007). Pulmonary rehabilitation: joint ACCP/AACVPR evidence-based clinical practice guidelines. Chest. 131(5 Suppl):4S-42S. (20)	361	Guideline
6	McCarthy B. (2015). Pulmonary rehabilitation for chronic obstructive pulmonary disease. Cochrane Database Syst Rev. (2):CD003793. (21)	335	Review
7	Borg GA. (1982). Psychophysical bases of perceived exertion. Med Sci Sports Exerc. 1982;14(5):377–81. (22)	303	Symposium Article
8	Lacasse Y. (2006). Pulmonary rehabilitation for chronic obstructive pulmonary disease. Cochrane Database Syst Rev. (4):CD003793. (23)	277	Review
9	Rabe KF. (2007). Global strategy for the diagnosis, management, and prevention of chronic obstructive pulmonary disease—GOLD executive summary. Am J Respir Crit Care Med. 176(6):532–555. (24)	270	Review
10	Holland AE. (2014). An official European respiratory society/American thoracic society technical standard: field walking tests in chronic respiratory disease. Eur Respir J. 44(6):1428–46. (25)	240	Guideline

Citation bursts can be analyzed by identifying references that researchers focused on during certain periods of time. In the present study, references with strongest citation bursts were confirmed by using CiteSpace V, and the minimum burst duration was set to 2 years. The node type was confined as “Cited Reference,” and the other parameters were set in accordance with the description in the Materials and Methods section. As shown in Figure 7B, burst strength values of the top 20 references with the strongest citation bursts ranged from 16.4024 to 91.1087. “Nici et al. (18), Am J Respir Crit Care, v173, p1,390” had the highest burst strength (91.1087), in addition, among the 20 references there were eight co-cited references which had recent bursts: “Mccarthy et al. (21), Cochrane Db Syst Rev,” “Holland et al. (25), Eur Respir J,” “Rochester et al. (9), Am J Respir Crit Care,” “Spruit et al. (16), Am J Respir Crit Care,” “Puhan et al. (26), Cochrane Db Syst Rev,” “Vogelmeler et al. (27), Am J Respir Crit Care,” “Holland et al. (28), Thorax,” and “Alisona et al. (29), Respirology,”. The major types of these co-cited references with recent bursts were guideline, review and randomized controlled trial.

### Analysis of Keywords

**Figure 8:**
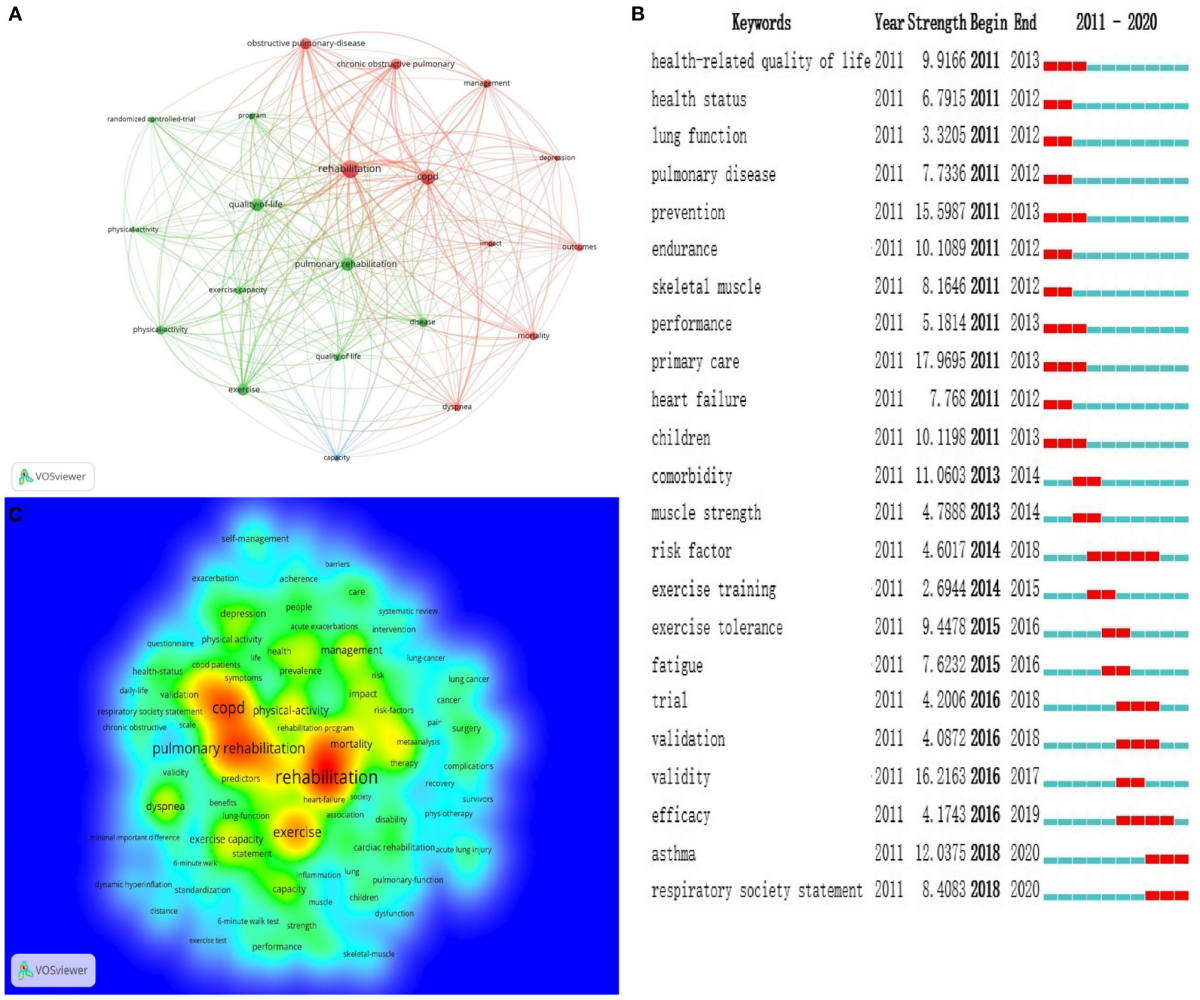
**(A)** Map of keywords co-occurrence related to pulmonary rehabilitation from 2011 to 2020 (T ≥ 200). **(B)** Top 20 Keywords with the Strongest Citation Bursts. **(C)** The density map of keywords, in which the closer the keyword node color is to red, the higher the frequency of its co-occurrence.

High-frequency keywords could reflect research hot spots (30). After calculation by VOSviewer, 10,747 keywords in total were extracted from all 4,521 publications. Ultimately, 21 high-frequency keywords with at least 200 occurrences were identified. The top 10 keywords with high Frequency and Centrality were shown in Table 6. “Rehabilitation” was the first frequency keyword, while “quality of life” was the first one with centrality. In addition, the keywords (T ≥ 200) were classified into three clusters of red, green and blue (Figure 8A). The red clusters are composed of “chronic obstructive pulmonary disease,” “copd,” “mortality,” “rehabilitation,” and “obstructive pulmonary disease.” The Green cluster included “disease,” “exercise,” “exercise capacity,” “physical activity,” “program,” “pulmonary rehabilitation,” and “quality of life.” According to the density map of keywords, “rehabilitation,” “COPD,” “pulmonary rehabilitation,” “mortality,” and “exercise” had higher weight (Figure 8C).

**Table 6:** Top 10 keywords with frequency and centrality related to pulmonary rehabilitation.

**Frequency**	**Keywords**	**Centrality**	**Keywords**
1,728	Rehabilitation	0.15	Quality of life
1,245	COPD	0.12	Mortality
1,069	Quality of life	0.09	Obstructive pulmonary disease
864	Pulmonary rehabilitation	0.08	Rehabilitation
849	Exercise	0.08	Exercise
764	Obstructive pulmonary disease	0.08	Program
571	Chronic obstructive pulmonary disease	0.07	Pulmonary rehabilitation
564	Physical activity	0.07	Capacity
519	Disease	0.07	Care
417	Mortality	0.06	Exercise capacity

Burst keywords, coming from popular keywords, refer to the high-frequency keywords that burst out at a certain stage, which can reflect the hot spot evolution of a research field and predict the research trend. Among popular keywords, there were 44 burst keywords with a high strongest citation, and Figure 8B listed the top 20 keywords with the strongest citation bursts. Furthermore, research frontiers history of PR between 2011 and 2020 can be drawn from the evolution of burst keywords used in articles. Obviously, “asthma” and “respiratory society statement” were the most recent ones, in which “asthma” had the highest strength of citation burst of 12.0375 since 2018.

## Discussion

### General Information

In the present study, we used bibliometric methods by CiteSpace and VOSviewer to analyze publications related to PR. The results showed that the number of annual publications on PR has shown an overall upward trend in the past decade, indicating that PR is getting more attention. Most notably, from 2019 to 2020, the literature increase was higher than what was presented in the previous periods. This may be related to the prevalence of Coronavirus disease 2019 (COVID-19). COVID-19 began at the end of 2019 and spread globally in 2020. As well as psychological therapy, PR is also crucial for COVID-19 survivors with the symptomatic burden of dyspnea and fatigue (31).

On the other hand, the results of this present study also visually showed that the regional characteristics of PR research were obvious, and developed countries were dominant in the research direction of PR. The USA was not only the most productive country but also the one leading citations, indicating that the USA was the core country in this field. But interestingly, the top 10 institutions came from Canada, the Netherlands, and Australia, containing not a single one from the USA. The University of Toronto in Canada was both the first in publications and citations, suggesting that the University of Toronto was the core institution in the field of PR. Moreover, international cooperation was mainly concentrated among developed countries. Access to PR is limited in many geographic areas especially the developing countries (9). As mentioned in the 2015 ATS/ERS statement, insufficient funding, limited resources and lack of knowledge and skill of healthcare professionals and patients regarding the benefits of PR are the main barriers to the implementation of PR (9). However, CRDs are global problems, and international cooperation, especially between developed and developing countries, is needed to improve the popularization and application of PR.

In addition, this present study showed Spruit MA was not only the most productive author but also the ranking first co-cited author, suggesting Spruit MA was the core researcher in the field of PR, who mainly focused on PR in CRDs (32). Highly productive and high co-cited authors have an important influence in this research field and promote the development of pulmonary rehabilitation. According to the analysis of journals and co-cited journals, we found that the first productive journal was the International Journal of Chronic Obstructive Pulmonary Disease (*n* = 209), while the first co-cited journal was the American Journal of Respiratory and Critical Care Medicine. Compared with top co-cited journals, the top 10 prolific journals were mostly divided into Q2 or Q3 with an average IF of 3.26, and their academic influence in this field is limited. Most notably, the SCR of the ranking first prolific journal was more than 10%. A journal's IF may be raised by increasing self-citation, however, self-citation was more commonly found in journals with a lower IF (33). Therefore, a prolific journal does not mean that it has a good academic influence, moreover, improving the quality of published research while increasing output may contribute to enhancing their academic influence (34).

References are vitally important for the selection, execution, and summary of scientific research (35), in addition, co-cited references with high co-citations have important academic influence in a certain field, while references with citation bursts suggest the focus of a certain field for a period of time. In the present study, the main type of co-cited references with top co-citations and citation bursts on PR were guidelines, reviews, and randomized controlled trials. Our analysis shows that guidelines, high-quality reviews, and randomized controlled trials may provide reliable evidence for more studies on PR.

## Research Hotspots and Trends

According to the top 10 frequency and centrality keywords (Table 6) and clustering analysis of keywords (Figure 8A), research hotspots on pulmonary rehabilitation mainly focused on the following aspects:

### Pulmonary Rehabilitation Improves the Exercise Capacity of Patients With CRD

Patients with CRDs often experience disabling symptoms (including dyspnea and fatigue) and exercise intolerance (9). Exercise capacity is considered an independent, potentially modifiable predictor of clinical outcomes in CRD (36). Therefore, improvement of exercise capacity has positive significance for judging the prognosis (37). Although PR includes but is not limited to exercise training, exercise training is considered the cornerstone of PR (18). Improvements in skeletal muscle function after exercise training, including respiratory muscle and peripheral muscle lead to gains in exercise capacity (16). Usually, Field walking tests, including 6-min walk distance (6MWD), incremental shuttle walk test (ISWT), and endurance shuttle walk test (ESWT), are used as evaluation indicators to evaluate exercise capacity (25). High-quality evidence showed that 6MWD and shuttle walk test distance improved by 62 and 48 m, respectively (26). In addition, pulmonary function, including forced expiratory volume in 1 s (FEV1) and forced vital capacity (FVC), and Symptoms such as dyspnea were also improved to varying degrees after PR (38, 39).

### Pulmonary Rehabilitation Improves Quality of Life of CRD

Affected by chronic lung disease, the quality of life of patients tends to decline, containing four important domains of quality of life [Chronic Respiratory Questionnaire (CRQ) scores for dyspnoea, fatigue, emotional function and mastery] (21). Studies showed that poor sleep quality is often reported in patients with CRD (40), as well as anxiety and depression (41). Mounting evidence showed that the quality of life of patients with CRD under the intervention of pulmonary rehabilitation was significantly better than that of non-pulmonary rehabilitation (42), which is mainly reflected in the improvement of symptoms and common evaluation indicators, such as Health-related quality of life (HRQoL), St. George's Respiratory Questionnaire (SGRQ), Hospital Anxiety and Depression (HAD), Short Form-36 (SF-36), and Pittsburgh Sleep Quality Index (PSQI).

### Effect of Pulmonary Rehabilitation on Mortality of CRD

Although the concept of PR was put forward as early as the 20th century, it has not been well-developed in the past few decades. An important reason for the slow development of PR is that its impact on the mortality of chronic lung disease is not clear. Although a great amount of evidence suggested that PR can bring clinically positive improvements in exercise capacity and quality of life, the effect of PR on mortality remains controversial (43). Notably, a rising number of studies have given positive results in reducing mortality. A Turkish study showed that the general annual mortality rates of patients with COPD who underwent PR were significantly lower than the ones without PR (44). Moreover, a systematic review and meta-analysis showed a moderate quality of evidence on reductions in mortality after early PR in patients hospitalized with a COPD exacerbation (45). However, due to the particularity of research on mortality, few large sample and high-quality studies have shown that PR can reduce the mortality rate of chronic lung disease. Further research is needed to overcome these limitations in the future.

## Research Frontiers

Burst keywords are detected from a large number of keywords and determine the development trend of a research field according to the changing trends of word frequency. Burst keywords with high strength at a certain stage represent the frontier field of this stage. In the present study, We captured the burst keywords by using CiteSpace. According to the analysis of burst keywords, two current frontiers of related research were identified as follows: asthma (2018–2020), and respiratory society statement (2018–2020). These keywords covered the research frontier of the current topic.

### Pulmonary Rehabilitation for Asthma

The prevalence of asthma remains at a high level worldwide, despite the development of drug therapy, the mortality rates have been static for decades (46). Therefore, in order to reach a comprehensive approach to disease management, the importance of non-pharmacological treatment in addition to pharmacological therapy has been recently highlighted. In the past decades, the evidence base for PR is the largest in the area of COPD, yet the level of evidence is lower in asthma (47, 48). As a heterogeneous disease, asthma always results in airflow limitation just like COPD. However, airflow limitation caused by COPD is incompletely reversible, while that caused by asthma is completely reversible. Global Initiative for Asthma (GINA) guidelines do not advocate PR as a part of clinical management, suggesting advice should be provided about PR for those with COPD or asthma—COPD overlap (49). A recent study showed that Pulmonary rehabilitation brought positive results on exercise tolerance, respiratory symptoms, and QoL in asthmatic patients at any step of the disease (50). The trial showed that a 3-week course of PR leads to clinically relevant improvement in asthma control and secondary endpoints. Patients who do not achieve control of their asthma despite outpatient treatment, therefore, benefit from rehabilitation (51).

### Respiratory Society Statement on Pulmonary Rehabilitation

After several decades of development, although mounting clinical evidence have shown that pulmonary rehabilitation plays a positive role in improving CRD, however, in fact, even in developed countries, the implementation status of PR is only <1.2% of people with COPD can get pulmonary rehabilitation intervention (52). Actually, the purpose of the respiratory society statement on PR is not only to evaluate clinical evidence within a period of time and update clinical practice guidelines, but also to promote the implementation and delivery of PR (53). The eventual aim of related statements from professional society is to expand the provision of PR to suitable patients with CRD all around the world. Since it was first written into the statement by ATS/ERS in 2006 (9), pulmonary rehabilitation is being recommended by more authoritative or influential academic groups. Meanwhile, the considerable growth in the science and application of PR increases further support for its efficacy in a great many patients with chronic respiratory disease.

### Advantages and Limitations

Compared with traditional reviews, the present study provides a better insight into research trends and frontiers in the field of PR based on bibliometric tools, simultaneously, data analysis on various aspects is relatively more comprehensive and objective. However, because of the limitations of software and research methods, the selected articles are limited to the ones included in the database of WOS-CC, whereas those included in other databases, such as PubMed and the database from China, have not been searched. Therefore, a total of 4,521 articles are not fully representative of all available information in this field, which is the major limitation of this article. Although we did not give a comprehensive analysis of every article related to the topic from all databases around the world, we still believe that this present study can provide a description of the research status and trends of pulmonary rehabilitation, for WOS-CC contains a large number of high-quality core journal papers all over the world.

## Conclusion

The trends of annual publications indicated that research on PR has received more attention. By using bibliometric analysis, the most active authors, countries, organizations, and journals were identified. Co-citation reference analysis revealed the top articles of great significance in this field. Popular keywords provide the research hotspots, while the burst keywords may provide the current research trends and frontiers. In summary, this study provides not only the research status and hot spots, but also the trends and frontiers of research on PR, and in some sense, it may provide some new directions for future research in this field.

## Author Contributions

TL was responsible for study design, data collection, statistical analysis, data interpretation, manuscript preparation, and literature search. JC took part in study design and data interpretation. All authors contributed to the article and approved the submitted version.

## Conflict of Interest

The authors declare that the research was conducted in the absence of any commercial or financial relationships that could be construed as a potential conflict of interest.

## Publisher's Note

All claims expressed in this article are solely those of the authors and do not necessarily represent those of their affiliated organizations, or those of the publisher, the editors and the reviewers. Any product that may be evaluated in this article, or claim that may be made by its manufacturer, is not guaranteed or endorsed by the publisher.
